# Managerial and environmental determinants of clinical mastitis in Danish dairy herds

**DOI:** 10.1186/1751-0147-50-4

**Published:** 2008-02-07

**Authors:** Kenji Sato, Paul C Bartlett, Lis Alban, Jens F Agger, Hans Houe

**Affiliations:** 1 Department of Veterinary Sciences, College of Agriculture, University of Wyoming, Laramie, WY, USA; 2 Department of Large Animal Clinical Science, Michigan State University, East Lansing, MI, USA; 3 The Danish Meat Association, Vinkelvej 11, DK-8620 Kjellerup, Denmark; 4 Department of Large Animal Sciences, Group of Population Biology, Section for Veterinary Epidemiology, Faculty of Life Sciences, University of Copenhagen, Grønnegårdsvej 8 DK-1870 Frederiksberg C, Denmark

## Abstract

**Background:**

Several management and environmental factors are known as contributory causes of clinical mastitis in dairy herd. The study objectives were to describe the structure of herd-specific mastitis management and environmental factors and to assess the relevance of these herd-specific indicators to mastitis incidence rate.

**Methods:**

Disease reports from the Danish Cattle Data Base and a management questionnaire from 2,146 herds in three Danish regions were analyzed to identify and characterize risk factors of clinical mastitis. A total of 94 (18 continuous and 76 discrete) management and production variables were screened in separate bivariate regression models. Variables associated with mastitis incidence rate at a p-value < 0.10 were examined with a factor analysis to assess the construct of data. Separately, a multivariable regression model was used to estimate the association of management variables with herd mastitis rate.

**Results:**

Three latent factors (quality of labor, region of Denmark and claw trimming, and quality of outdoor holding area) were identified from 14 variables. Daily milk production per cow, claw disease, quality of labor and region of Denmark were found to be significantly associated with mastitis incidence rate. A common multiple regression analysis with backward and forward selection procedures indicated there were 9 herd-specific risk factors.

**Conclusion:**

Though risk factors ascertained by farmer-completed surveys explained a small percentage of the among-herd variability in crude herd-specific mastitis rates, the study suggested that farmer attitudes toward mastitis and lameness treatment were important determinants for mastitis incidence rate. Our factor analysis identified one significant latent factor, which was related to labor quality on the farm.

## Background

Mastitis is defined as an inflammation of the parenchyma of mammary gland, regardless of the specific etiologic agent [1]. Clinical mastitis (CM) is known to be caused by several bacterial pathogens such as *Streptococcus agalactiae*, *Staphylococcus aureus*, *E. coli *and mycoplasma, however, the presence of pathogens in the mammary gland is often not sufficient to cause CM. It is generally believed that management and environmental factors are important contributory causes of CM. Factors such as housing [2], nutrition [3,4], milk production, milking procedures [5], and dry cow treatment [6] have been found to be associated with CM incidence.

Many epidemiological studies have examined herd-specific management and environmental risk factors; however most of these studies were conducted on a small number of herds. Because the herd must be the unit of observation for such studies, a large database is required to adequately estimate the effect of herd-specific management and environmental factors. Collecting reliable information on management factors and herd-specific rates of CM can be difficult and expensive, and has limited the size of many previous studies. Another difficulty is that many management variables are strongly interrelated, creating potential collinearity problems for statistical analysis

Direct management causes of CM may be difficult to measure on a management survey. For example, a questionnaire may collect data regarding the use of hired help (an indirect cause), but the more direct cause of mastitis might really be a poor proficiency in milking technique and motivation to follow proper procedures. Another difficulty encountered when studying CM risk factors is that risk factors for one mastitis etiologic agent, e.g. *Staphylococcus aureus *mastitis, may be different from the risk factors for mastitis caused by other etiologies, e.g., coliform mastitis. While acknowledging these considerations, delineation of the major CM risk factors by large observational studies is an important first step in characterizing the causal factors associated with reported CM in different geographies. Designed and controlled field trials will eventually be required to further evaluate the causal importance of specific risk factors for specific CM etiologic groupings.

This current study focused on herd-specific management and environmental factors related to CM incidence in the Danish dairy industry. The study objectives were to describe the structure of herd-specific mastitis management and environmental factors and to assess the relevance of these herd-specific indicators to mastitis incidence rate.

## Methods

### Data

**Figure 1:**
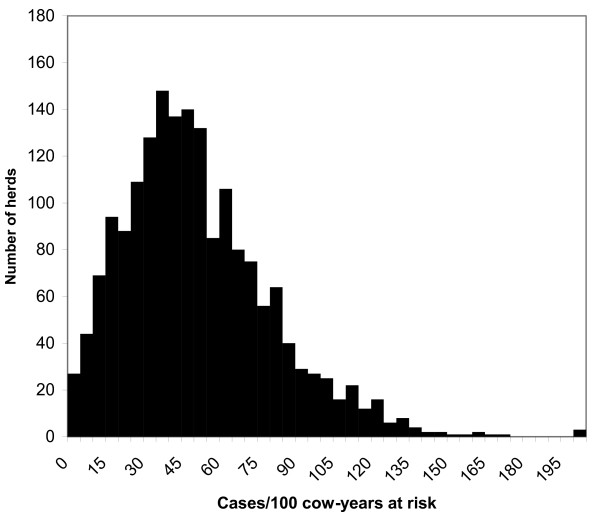
Frequency distribution of the herd mastitis incidence rates. Label on X-axis: Cases/100 cow-years at risk. Label on Y-axis: Number of herds.

The data from the Danish Cattle Data Base and a management questionnaire from 2,146 herds in three Danish regions were used for this study [7-9]. The sample of herds constituted 20% of the Danish dairy herd population in 1993. Specific codes in the Danish Cattle Data Base for various types of mastitis (acute mastitis, mastitis secondary to teat lesion, necrotic, unspecified mastitis, summer mastitis, and mastitis in a dry cow) were all combined into a single "clinical mastitis" category for this study. The majority of antibiotic treatments were probably initiated by the local veterinarians in Denmark [10], however farmers probably often used medicines left by the veterinarian to complete treatment protocols in subsequent days following the veterinarian's visit. The herd-specific mastitis incidence rates were calculated over a one-year period from July 1, 1993 to June 30, 1994, with the management survey being conducted at the end of this period [11]. The data from all 2,146 herds were screened for evidence of non-reporting behavior. Each herd was visited 11–12 times annually by a milk tester, at which time a somatic cell count (SCC) determination was obtained for each lactating cow. In order to identify non-reporting behavior, we identified cows with SCC tests of over 1,000,000 cells/*ml *and determined the herd percentage of these cows that had a CM report within 30 days before this high SCC test result. If this percentage was below 2% for a particular herd, the herd was suspected of non-reporting behavior and was removed from the current analysis. On this basis, 1,800 of the original 2,146 herds remained for study [11]. After removal of expected non-reporting farms, the frequency distribution of the herd CM incidence rates represented a reasonably normal distribution (Shapiro-Wilk statistic = 0.934; figure 1). The CM incidence rate was calculated as: (number of cows with CM during the year/total number of cow-days at risk) × 365 days per year × 100 cows. Individual cows with CM did not accumulate days at risk after their first reported mastitis cases. A total of 68,788 mastitis cases in 56 million cow-days-at-risk were identified for this analysis.

A management questionnaire was designed to obtain information on housing, grazing, work load, replacement of animals, and procedures for prevention, treatment and recording of disease. This questionnaire included a total of 94 (18 continuous and 76 discrete) management and production variables [7-9].

### Statistical analysis

As the first step of our analysis, all explanatory variables were individually screened in separate simple regression models as predictors of CM. (PROC GLM in the SAS statistical software). Data on farmer's speculation or opinion (i.e. How satisfied with cow health: SUNDH_51, How satisfied with cow welfare: VELF_52 and Intensity of cow house: BELAEG65) were excluded from the final multivariable model.

#### Factor analysis

Since many management variables in the questionnaire were strongly correlated with each other, ordinary statistical test were inadequate. In response, factor analysis was chosen as a way of dealing with multicollinearity. Factor analysis is a technique that was originally developed to understand the link between student performance measured in terms of grades and intelligence. Hence, this technique enables the measurement of an underlying construct that can be separated into one, two or more dimensions [12]. In our case, we used factor analysis to identify the smallest number of common factors that best explains the correlations among the indicators, describing management in dairy herds. The number of factors to extract is subjective; guidelines exist and are based on the scree-plot (find the elbow of the plot) and the amount of variance that the factors explain in total. A factor consists of several variables; where those with highest loadings are most influential. Based on the influential variables an interpretation of the factor can be made. For example, the influential variables indicate, that a factor deals with quality of labor. Here, a high score on this factor corresponds to a high quality of the labor, and similarly a low score corresponds to a low quality of labor. For a more detailed description of factor analysis, please see Sharma [12].

Only variables significantly associated with herd CM at p < 0.10 in the Type I test of simple regression analyses were used for the factor analysis. Common factor analysis in line with an inspection of the scree-plot was performed to determine how many latent factors could be found among the independent variables. Because there were nominal, ordinal and continuous variables, the PRINQUAL (principal components of qualitative data) procedure with maximum total variance method was used to obtain the correlation matrix. The PRINQUAL procedure is a data transformation procedure that enables nominal and ordinal variables to have optimized covariance or correlation matrix for the following factor analysis. The correlations of each variable with all other variables were used as prior communality estimates in the factor analysis (PROC FACTOR). The principal factors method was used for the extraction of the factors [13]. Factors that accounted for over 10% of the common variance were selected as the final factor solution. Variables with high loadings were identified for each factor. The factors were tested as predictors of CM rates with the general linear model (PROC GLM).

#### Multivariable and the following analysis

Variables with p < 0.25 in the simple regression analysis were used for constructing a multivariable regression model to explain herd CM. A backward and a forward selection procedure based on the F test statistic were conducted to select variables for inclusion [14]. The diagnostic criterion for CM (SLEMT_57) was forced into the model to help control reporting bias with regards to how each farmer defined a case of CM. Variables with p < 0.05 were retained in the final model. All pair-wise combinations of variables were evaluated for the possible interaction and the residuals were closely monitored for normality during all model-building steps.

All statistical analysis was performed using SAS statistical software (version 8.02; SAS Institute, Cary, NC).

## Results

Mastitis cases were reported to the Danish Cattle Data Base by veterinarians (77%) and by producers (23%), with duplicate reports being eliminated [11]. The grand mean rate of CM was 44.7 cases per 100 cow-years at risk (median = 41.0, Shapiro-Wilk statistic = 0.935). A total of 34 management variables with P-value < 0.25 in the bivariate test of association with CM are shown in [see Additional file 1].

### Factor analysis

**Figure 2:**
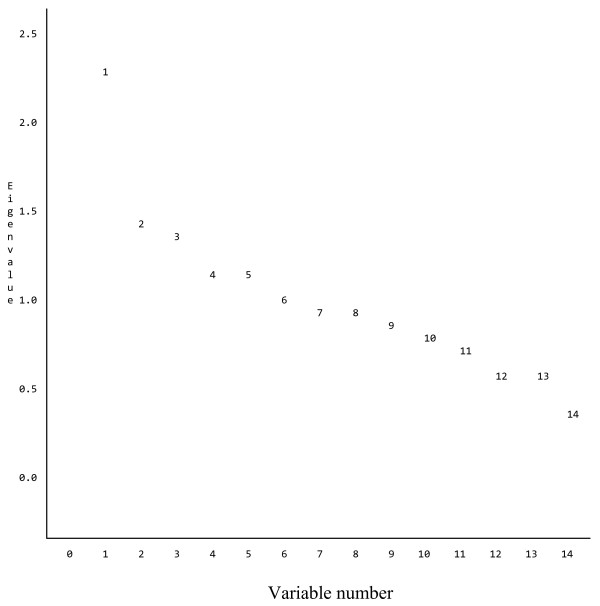
Scree plot of Eigenvalues for 14 variables. Label on X-axis: Variable number, Label on Y-axis: Eigenvalue, Legends: Numbers inside the figure indicate the variable number.

Fourteen variables were found to have p-values < 0.10 in the initial bivariate analysis, and these variables were subjected to an exploratory factor analysis. The highest correlation was 0.61 between "who takes care of the cows?" (PASSER_1) and "hired labor used in cow house?" (FHJLP_73). The second and third highest squared correlations were 0.51 and 0.43 between PASSER_1 and TILSYN32, and between TILSYN32 and FHJLP_73 respectively. All other squared correlations were below 0.35. The eigenvalues of the correlation matrix (not shown in this report) showed that the percentage of common variance accounted for by factor 1, 2, and 3 were 16%, 10% and 10%, respectively. The factors 4 to 14 each accounted for less than 10% of the common variance. If we choose the number of latent factors based on eigenvalues greater than 1 (the Kaiser criterion [15]), there would be 6 latent factors for this data. The scree plot of the eigenvalues (Figure 2) shows a sizable gap between the factors with relatively large eigenvalues (factor 1–3) and those with smaller eigenvalues (factor 4–14). Therefore, three factors were contained in the model.

The rotated factor pattern is shown in Table 1. High loadings were observed with "who takes care of cows (PASSER_1), "who manages the cows" (TILSYN32), "hired labor used in cow house" (FHJLP_73), and "milking and feeding man-hours per cow" (ARB3031) for factor 1, "Region of Denmark" (OMR), "Shelter available on pasture" (LAEM_22), "which cows are trimmed" (KLOVH_64) and "what percentage of the cows had claw diseases during the recent year" (UKLOV_43) for factor 2 and "Shelter available on pasture" (LAEM_22) and "Do cows get stone bruises in the claws" (STEN_29) for factor 3. A general regression analysis on association between CM incidence rate and factor 1–4 showed that only factor 1 was highly associated with CM rate (p < 0.0001).

**Table 1:** Varimax Rotated Factor Pattern Matrix. The matrix represents standardized regression coefficients for predicting the variables using the extracted factors.

Variables	CODE	Loadings		
		
		Factor 1	Factor 2	Factor 3
Region of Denmark	OMR	5	77*	-8
Who takes care of the cows?	PASSER_1	85*	-2	7
Age of cow house floor	ALDGM_12	-11	2	2
Shelter available on pasture	LAEM_22	-19	-30*	74*
Do cows get stone bruises in the claws?	STEN_29	-12	22	81*
Who manages the cows?	TILSYN32	74*	-13	4
How do you handle cows with mastitis apart from the veterinary treatment?	YVSYG_38	9	2	11
Which cows are trimmed?	KLOVH_64	10	46*	-10
Hired labor used in cow house	FHJLP_73	-82*	-5	-5
Milking and feeding man hours per cow	ARB3031	42*	12	-8
Average daily milk production per cow	MILK	10	-22	1
Approximate what percentage of the cows had claw diseases during the recent year	UKLOV_43	5	-60*	-16
How do you decide to cull a cow ^(1)^	GRUDS_71	6	0	15
Age of dairy producer	ALDER_76	-9	3	-7

Printed loadings are multiplied by 100 and rounded to the nearest integer.

* value greater than 0.3

^(1) ^Herds where cows were culled due to udder disease were compared with herds where cows were not culled due to udder problems. Udder disease included mastitis and other udder problems.

### Multivariable Regression Model

A total of 9 variables were retained in the final multivariable regression model (Table 2). Herd CM incidence rate was significantly lower (42.8 vs. 48.0) among farmers who only reported CM cases to the Danish Cattle Data Base when they noticed abnormality in both milk and gland, justifying our decision to include diagnostic criteria in the multiple regression model to control what otherwise could have been a reporting bias. Regions 7 & 9 (Funen and SW Jutland) of Denmark were highly associated with high CM incidence rate, which may have been due to differences in reporting behavior, management factors or environmental differences among the regions. Antibiotic use in "How do you handle cows with mastitis apart from the veterinary treatment" (YVSYG_38) was associated with lower incidence of mastitis, however only a few farms (35) reported the use of antibiotics. The mastitis incidence rate was increased when the proportion of claw disease cow was increased (UKLOV_43). Farms where only cows with claw problems had their hooves trimmed had significantly lower mastitis incidence rate than farms where all cows or no cows were trimmed (KLOVH_64: p = 0.0033). All pair-wise combinations of variables and quadratic terms were evaluated for the possible interaction in the final model, however none were significant.

**Table 2:** Final General Linear Model for the incidence rate of CM.

**Variable Description**	**Code**	**Values**	**Correlation coefficient estimate**	**Type III p-value**
Region of Denmark	OMR	Region 5	-5.652	<.0001
		Region 7	-0.196	-
		Region 9	-	-
Who takes care of the cows?	PASSER_1	Husband	-3.349	0.0145
		Wife	-	-
Age of cow house floor	ALDGM_12	Less then 20	-3.907	0.0015
		Over 20 years	-	
How do you handle cows with mastitis apart from the veterinary treatment	YVSYG_38	Use Antibiotics	-11.612	0.0086
		Do nothing	-	
How do you handle lame cows apart from veterinary treatment	KLOVL_42	Trim & Clean	2.843	0.0308
		Do nothing	-	
Diagnostic criteria for CM ^(1)^	SLEMT_57	milk & gland	-5.491	<.0001
		milk only	-	
Which cows are trimmed	KLOVH_64	Trim selected cow	-4.140	0.0033
		Trim all cow	-	
Approximate what percentage of the cows had claw diseases during the recent year ^(2)^	UKLOV_43	% of cows with claw disease	0.367	0.0010
Daily milk production per cow ^(3)^	MILK	Kg/day/cow	1.256	<.0001

(1) Diagnostic criteria was forced into the multivariable analysis

(2) When claw disease increased by 10%, the mastitis incidence rate was increased by 3.67 cases/100-cow years.

(3) When milk production increased by 10 kg, the mastitis incidence rate was increased by 12.56 cases/100-cow years.

## Discussion

The limitation of field surveillance data regarding mastitis treatments were discussed in a previous publication [11]. It is recognized that farmers and veterinarians used different diagnostic criteria regarding when mastitis clinical signs were sufficiently severe to warrant antibiotic treatment by their veterinarian. The disease reports from the Danish Cattle Data Base are based upon those diagnostic criteria the farmer used to decide whether or not to call the veterinarian, and the diagnostic criteria the veterinarian used regarding which cows required treatment. As such, our case definition includes those cases of mastitis that were sufficiently severe that the farmers and their veterinarians decided to take therapeutic action and these criteria may be very different from herd to herd [16].

Danish law mandates that all antibiotics were to be given under the direction of a veterinarian and therefore all antibiotic mastitis treatments should have been reported in the database. However, at that time there was no legal requirements to report the treatments to the database even though the Danish Veterinary Association required this of their members. Certainly under-reporting occurred, but the extent of the under-reporting is unknown. While there were 35 farms that reported administering antibiotics "apart from the veterinary treatment", some farmers may have considered the "veterinary treatment" to have been limited to the drugs administered by the veterinarian on the day of the farm visit and not to have included the drugs left behind for administration by the farmer. Also, mastitis drugs leftover from previous treatments may have been administered at the initiative of the farmer. Some farmer-initiated non-antibiotic treatments may also have been administered. The instances of illegal, non-veterinary administration of antibiotics to cattle were probably very rare.

We did not detect significant associations with some previously identified risk factors such as herd size, amount of bedding or type of bedding materials. This was in contrast to a previous study in Ohio, USA [17-20]. This may be because of differences between Denmark and the USA, but may also be because the crude herd rate of CM in Denmark represents the composite of many contributory clusters of mastitis causation with different etiological agents. Because the dependent variable in the current study represented a summation of CM incidence due to many different ecological systems (webs of causation), our ability to accurately predict crude herd-level rate is understandably low. Barkema et al. [4] studied agent-specific CM rates and reported herd-specific risk factors for mastitis caused by *E. coli*, *Staphylococcus aureus*, *Streptococcus dysgalactiae*, and *Streptococcus uberis*. Housing conditions, hygiene, and machine milking were found to be associated with *E. coli *mastitis; whereas nutrition and milking technique were more important for *Streptococcus dysgalactiae*.

Although farmers' perceptions of general cow health (SUNDH_51), welfare of cows (VELF_52), and use intensity of cow house (BELAEG65) were significantly associated with mastitis incidence rate, these variables were excluded from the factor analysis and multivariable analysis because we reasoned that these variables could be both determinants and consequences of increased CM rates. Also, two variables (dry cow treatment with antibiotics (GOLDA_50), and culling cows due to udder problems (GRUDS_71) were excluded from the multiple regression model since these variable were considered attempted interventions for an increased CM rate rather than being suspected risk factors in the causation of CM. As is always the case with observational studies, variables found to be significantly associated with the incidence rate of CM do not necessarily indicate a causal relationship, but suspected effects of disease should be excluded from evaluation as possible risk factors.

### Factor analysis

The purpose of factor analysis in general is to discover possible simple latent factors based on the correlations between the numerous variables obtained by questionnaire. However, this heuristic analysis would not provide a definitive number of latent factors underlying the management, and involved somewhat subjective decisions involving issues such as how many factors should be retained. If we use Kaiser criterion (eigenvalue >1) to determine the number of latent factors, it retains too many factors (six), while if we use scree test, it retains only few factors. Communality is the proportion of variance in that variable which is explained by common factors. Communalities generally increase with number of latent factors. But the communalities are not used to choose the final number of latent factors. Low communalities are not interpreted as evidence that the data fail to fit any hypothesis, but merely as evidence that the variables analyzed have little in common with one another.

Though we evaluated one to six latent factors and tested association with CM in each case, only the latent factor 1 was significantly associated with CM rate. The remaining factors are expected to describe other aspects of cow health and production, reflecting the wide-range of questions that were covered by the questionnaire. The result indicated that each variable obtained by questionnaire were relatively unique to each other and combination of variables had higher predictability (R-square = 0.06) than those latent factors (R-square = 0.03). At the same time, the factor analysis clearly demonstrated that there was redundancy in the questionnaire, which was difficult to recognize without the factor analysis. For example, PASSER_1, TILSYN32, FHJLP_73 and ARB3031 shared latent factor 1. It appears that farms tended to use more hired laborers (FHJLP_73) if the main cow caretaker was the wife (PASSER_1) and the hired labors tend to spend more time for milking and feeding per cow (ARB3031). These variables could have been cause of collinearity if they were included in the regression equation at the same time. Factor 1 indicated that the data from the questionnaire consisted of a latent factor related to quality of labor.

### Multivariable Regression Model

Although only the first case of CM in a cow was included in the study, treating mastitis cows with an antibiotic apart from the veterinary treatment (YVSYG_38) was associated with a lower CM incidence rate (32.3 vs. 45.0). This association could be explained in that antibiotic use may prevent pathogens from spreading in the herd or it may reflect the general proactive attitude of the producer toward disease prevention. However, it is more probable that treatment without the involvement of a veterinarian resulted in reduced rates of mastitis reporting. Vaarst, et al. [16] qualitatively studied farmers' decision on antimicrobial use for mastitis and analyzed at four levels (mastitis symptoms, single-cow characteristics, the situation of herd and existing alternatives) of the decision-making process. They found that farmers were coherent in their choices of treatment, but their decisions were often different from their veterinarian's recommendations.

Higher producing herds had significantly higher rates of mastitis. This is consistent with other epidemiological studies in Europe and the U.S.A. [21,22] in which CM is seen as a 'production disease' because it is associated with high milk production. However, Kornalijnslijper et al. [23] concluded that host resistance to experimentally induced *E. coli *mastitis was not affected by the production level. Also, better management can lead to both high production and complete records of disease occurrence, which would then produce a non-causal, but positive association between milk production and rates of CM. The region of Denmark was an important determinant of herd CM rate, however the biological explanation for this association was not revealed in our study. One of the researchers recognized that region 5 consisted of more Jersey than Holstein herds, and suspected a lower mastitis rate in Jersey than in Holstein cattle [24]. The breed, which was not included in this analysis, could certainly have been a confounding factor.

Our study indicated that mastitis was associated to claw disease; however, claw disease is known to be associated to parity, stage of lactation, milk yield and other environmental factors [25,26]. Undefined common causes may increase rates of both claw disease and CM. Farmers who selectively treat cows with claw problems may pay more attention to individual cows, which resulted in lower mastitis incidence.

The R-squared value measures the percent of variability (total sum of squares) in the dependent variable that could be explained by the independent variables. The R-square for our multivariable models was very low (R-square = 0.06), indicating that most of the variability in herd mastitis rate could not be explained by the independent variables that we measured on our management questionnaire. Certainly our model may have been more predictive if we had include farmer's perception (observation on cow health, welfare and intensity of cow house), and if the investigators been able to personally visit each farm and directly observe the facilities and management procedures. Questionnaires completed by dairy producers often reflect intended procedures rather than actual procedures. While producer questionnaires can assess different management types, such as use of free stalls or tie stalls, it is impossible for dairy producers to judge the quality of their own management or the degree of skill or care with which a given procedure or management system is employed. Factors such as sanitation or milking hygiene cannot be assessed by the dairy producers themselves, since such assessments are intrinsically comparative and subjective. Such factors must necessarily be measured by investigator visits to the farm. Due to the large number of herds in the current study, investigator farm visits were not possible for the current study. The availability of information relating to milking hygiene and milking procedure would almost certainly have improved our ability to predict the herd mastitis rate. Also, studies of agent-specific mastitis rates would probably also increase R-squared values, as previously discussed.

## Conclusion

Though risk factors ascertained by farmer-completed surveys explained a small percentage of the among-herd variability in crude herd-specific mastitis rates, the study suggested that farmer attitudes toward mastitis and lameness treatment were important determinants for mastitis incidence rate. Our factor analysis identified one significant latent factor, which was related to labor quality on the farm. The General Linear Model indicated that dairy milk production per cow, claw disease, quality of labor and region of Denmark were significantly associated with mastitis incidence rate. Investigators' farm visits to measure factors such as quality of sanitation or milking hygiene could improve the CM risk analysis. Risk factor analysis would also likely be improved by analyzing agent-specific rates of mastitis rather than overall, composite or crude CM rates that undoubtedly include the effects of many independently operating causal pathways.

## Authors' contributions

KS carried out data analysis, interpretation, drafting the manuscript, and coordination among authors. PB conceived of the study, contributed data acquisition, conception, verification of analysis, interpretation of data and revising the manuscript. LA participated in the statistical design and analysis, and revising the manuscript. JFA and HH made substantial contribution to conception and revising the manuscript for important intellectual content in detail.

## Supplementary Material

Additional File 1 Results of selected simple regression analysis between herd level mastitis incidence rate and managerial and environmental variables using PROC GLM (P < 0.25). Click here for file

## References

[B1] Radostits OM, Gay CC, Blood DC, Hinchcliff KW, Arundel JH (2000). Mastitis, Veterinary Medicine.

[B2] Østeras O, Lund A (1988). Epidemiological analyses of the associations between bovine udder health and housing. Prev Vet Med.

[B3] Smith KL, Harrison JH, Hancok DD, Todhunter DA, Conrad HR (1984). Effect of vitamin E and selenium supplementation on incidence of clinical mastitis and duration of clinical symptoms. J Dairy Sci.

[B4] Barkema HW, Schkken Y, Lam TJGM, Beiboer ML, Benedictus G, Brand A (1999). Management Practices Associated with the Incidence Rate of Clinical Mastitis. J Dairy Sci.

[B5] Schukken YHF, Grommers FJ, van de Geer D, Erb HN, Brand A (1990). Risk factors for clinical mastitis in herds with a low bulk milk somatic cell count. 1. Data and risk factors for all cases. J Dairy Sci.

[B6] Berry EA, Hillerton JE (2002). The Effect of Selective Dry Cow Treatment on New Intramammary Infections. J Dairy Sci.

[B7] Alban L, Agger JF (1996). Welfare in Danish dairy herds 1. Disease management routines in 1983 and 1994. Acta Vet Scand.

[B8] Alban L, Agger JF (1996). Welfare in Danish dairy herds 2. Housing systems and grazing procedures in 1983 and 1994. Acta Vet Scand.

[B9] Agger JF, Alban L (1996). Welfare in Danish dairy herds 3. Health management and general routines in 1983 and 1994. Acta Vet Scand.

[B10] Bennedsgaard TW, Enevoldsen C, Thamsborg SM, Vaarst M (2003). Effect of Mastitis Treatment and Somatic Cell counts on Milk Yield in Danish Organic Dairy Cows. J Dairy Sci.

[B11] Bartlett P, Agger JF, Houe H, Lawson LG (2001). Incidence of clinical mastitis in Danish dairy cattle and screening for non-reporting in a passively collected national surveillance system. Prev Vet Med.

[B12] Sharma S (1996). Applied Multivariate Techniques.

[B13] Hatcher L (1994). A Step-by-Step Approach to Using SAS for Factor Analysis and Structural Equation Modeling.

[B14] Kleinbaum D, Kupper LL, Muller KE (1988). Applied Regression Analysis and Other Multivariable Methods.

[B15] Gorsuch RL (1983). Factor Analysis, Second Edition.

[B16] Vaarst M, Paarup-Laursen B, Houe H, Fossing C, Andersen HJ (2002). Farmers' choice of medical treatment of mastitis in Danish dairy herds based on qualitative research interviews. J Dairy Sci.

[B17] Bartlett PC, Miller GY, Lance SE, Heider LE (1992). Clinical mstitis and intramammary infections on Ohio dairy farms. Prev Vet Med.

[B18] Bartlett PC, Miller GY, Lance SE, Heider LE (1992). Managerial Determinants of Intramammary Coliform and Environmental Streptococci Infections in Ohio Dairy Herds. J Dairy Sci.

[B19] Bartlett PC, Miller GY, Lance SE, Heider LE (1992). Environmental and managerial determinants of somatic cell counts and clinical mastitis incidence in Ohio dairy herds. Prev Vet Med.

[B20] Bartlett PC, Miller GY, Lance SE, Heider LE, Anderson CR (1992). Environmental and managerial risk factors of intramammary infection with coagulase-negative staphylococci in Ohio dairy herds. Prev Vet Med.

[B21] Fleischer P, Metzner M, Beyerbach M, Hoedemaker M, Klee W (2001). The relationship between milk yield and the incidence of some diseases in dairy cows. J Dairy Sci.

[B22] Gröhn YT, Eicker SW, Hertl JA (1995). The association between previous 305-day milk yield and disease in New York state dairy cows. J Dairy Sci.

[B23] Kornalijnslijper E, Beerda B, Daemen I, van der Werf J, van Werven T, Niewold T (2003). The effect of milk production level on host resistance of dairy cows, as assessed by the severity of experimental Escherichia coli mastitis. Vet Res.

[B24] Washburn SP, White SL, Green JT, Benson GA (2002). Reproduction, mastitis, and body condition of seasonally calved Holstein and Jersey cows in confinement or pasture systems. J Dairy Sci.

[B25] Enevoldsen C, Gröhn YT, Thysen I (1991). Heel Erosion and Other Interdigital Disorders in Dairy Cows: Associations with Season, Cow Characteristics, Disease, and Production. J Dairy Sci.

[B26] Hultgren J, Manske T, Bergsten C (2004). Associations of sole ulcer at claw trimming with reproductive performance, udder health, milk yield, and culling in Swedish dairy cattle. Prev Vet Med.

