# Supplementary material for: Managerial and environmental determinants of clinical mastitis in Danish dairy herds
Source: Acta Vet Scand. 2008 Feb 7;50(1):4. doi: 10.1186/1751-0147-50-4 (PMC2268923; doi:10.1186/1751-0147-50-4)
Supplement: Additional File 1 — Results of selected simple regression analysis between herd level mastitis incidence rate and managerial and environmental variables using PROC GLM (P < 0.25). [file 1751-0147-50-4-S1.doc]

Results of selected simple regression analysis between herd level mastitis incidence rate and managerial and environmental variables using PROC GLM (P < 0.25).

| **Variable Description** | **Variable code** | **Values** | **N** | **Incidence rate** | **P** |
| --- | --- | --- | --- | --- | --- |
| **A. Basic Epidemiologic Parameters** |  |  |  |  |  |
| Region of Denmark | OMR | Region 5 | 883 | 41 | <.0001 |
|  |  | Region 7 | 285 | 47 |  |
|  |  | Region 9 | 632 | 47 |  |
| Herd Size | SG | ≤ 25 | 103 | 46 | 0.9451 |
|  |  | 25 < and ≤ 50 | 458 | 45 |  |
|  |  | 50 < and ≤ 75 | 571 | 44 |  |
|  |  | 75 < and ≤100 | 377 | 45 |  |
|  |  | >100 | 291 | 44 |  |
| Average Daily Milk Production Per Cow | MILK | Continuous variable | - | 1.24^(1)^ | <.0001 |
|  |  |  |  |  |  |
| **B. Physical Facilities** |  |  |  |  |  |
| Type of tie system. | BIND_4C | Not a tie system | 232 | 44 | 0.1255 |
|  |  | Neck bar | 909 | 43 |  |
|  |  | Chain | 215 | 48 |  |
|  |  | Yoke | 134 | 42 |  |
|  |  | Mixed | 310 | 46 |  |
| Walking area in loose housing | GANG_5B | Not loose housing | 1573 | 44 | 0.189 |
|  |  | Concrete or slats | 192 | 42 |  |
|  |  | Deep bed and other | 35 | 50 |  |
| Floor in bedding area | UNDERM_6 | No answer | 29 | 36 | 0.157 |
|  |  | Concrete | 1566 | 45 |  |
|  |  | Mats | 142 | 41 |  |
|  |  | Mix | 11 | 41 |  |
|  |  | Deep bed | 52 | 49 |  |
| Age of cow house floor | ALDGM_12 | < 20 years old | 933 | 43 | 0.0044 |
|  |  | 20 or more years | 867 | 46 |  |
| How many months are cows pastured per year | MGRAES27 | continuous variable | - | -0.31^(2)^ | 0.1905 |
| Shelter available on pasture | LAEM_22 | Not on Pasture | 357 | 46 | 0.063 |
|  |  | Not available | 243 | 41 |  |
|  |  | Available | 1200 | 44 |  |
| Distance between cow house to pasture | AFST_28 | < 50 meters | 933 | 43 | 0.1604 |
|  |  | 50 ≤ meters | 867 | 45 |  |
| Do cows get stone bruises in the claws | STEN_29 | Never | 529 | 43 | 0.0754 |
|  |  | Seldom or often | 1271 | 45 |  |
| **C. Labor quality** |  |  |  |  |  |
| Who takes care of the cows? | PASSER_1 | Husband | 1295 | 43 | 0.0041 |
|  |  | Wife | 505 | 47 |  |
| Who manages the cows | TILSYN32 | Only family | 1306 | 43 | 0.0602 |
|  |  | Family and laborer(s) | 354 | 46 |  |
|  |  | Manager involved | 140 | 48 |  |
| Same person always take care of the cows | SAMPER33 | No | 106 | 40 | 0.1088 |
|  |  | Yes | 1694 | 45 |  |
| Hired labor used in cow house. | FHJLP_73 | Constantly | 698 | 47 | 0.005 |
|  |  | Periodically | 230 | 44 |  |
|  |  | Never | 872 | 42 |  |
| **Farm Ownership** |  |  |  |  |  |
| Age of dairy producer | ALDER_76 | < 36 | 400 | 47 | 0.0035 |
|  |  | 36 ≤ and < 46 | 474 | 41 |  |
|  |  | 46 ≤ and < 54 | 447 | 45 |  |
|  |  | 54 ≤ | 479 | 45 |  |
| **Indicators of humane care** |  |  |  |  |  |
| Cow shearing description | KLIPK_60 | Not sheared | 462 | 43 | 0.2078 |
|  |  | Partly | 299 | 47 |  |
|  |  | Completely | 1039 | 44 |  |
| Which cows are trimmed (all, cows with claw problem) | KLOVH_64 | All cow | 1278 | 46 | <.0001 |
|  |  | Selected cows | 522 | 40 |  |
| How is water supplied outdoor | VANDU_67 | use drinking bowls | 64 | 40 | 0.1925 |
|  |  | other | 1736 | 44 |  |
| Frequency of checking water bowls. | VANDK_69 | Not known | 106 | 44 | 0.2233 |
|  |  | ≤ once per year | 465 | 47 |  |
|  |  | 2 ≤ and ≤ 11 per yr. | 341 | 43 |  |
|  |  | 12 ≤ | 888 | 43 |  |
| **Management Procedures** |  |  |  |  |  |
| Amount of bedding (kg) per cow. | STROEM_7 | < .4 | 335 | 41 | 0.2200 |
|  |  | 0.4 ≤ and < 1 | 390 | 45 |  |
|  |  | 1 ≤ and < 1.9 | 524 | 44 |  |
|  |  | 1.9 ≤ | 460 | 45 |  |
| Type of straw. | SNITM_8 | No straw | 156 | 40 | 0.0817 |
|  |  | Chopped | 904 | 45 |  |
|  |  | Not chopped | 740 | 44 |  |
| Milking and feeding man hours per cow | ARB3031 | < 2.75 min. | 449 | 42 | 0.1397 |
|  |  | 2.75 ≤ and < 3.2 min. | 438 | 45 |  |
|  |  | 3.2 ≤ and < 3.75 min. | 409 | 44 |  |
|  |  | 3.75 ≤ minutes | 501 | 46 |  |
| Approximate what percentage of the cows had claw diseases during the recent year | UKLOV_43 | continuous variable | - | 0.36^(3)^ | 0.0014 |
|  |  |  |  |  |  |
| How do you decide to cull a cow | GRUDS_71 | udder disease | 1158 | 47 | <.0001 |
|  |  | other reason | 642 | 40 |  |
| **Medication** |  |  |  |  |  |
| How do you handle cows with mastitis apart from the veterinary treatment | YVSYG_38 | Treat with antibiotics | 35 | 32 | 0.0051 |
|  |  | No antibiotics used | 1765 | 45 |  |
| How do you handle lame cows apart from veterinary treatment | KLOVL_42 | Trim or clean | 1187 | 45 | 0.1332 |
|  |  | Do nothing | 613 | 43 |  |
| Who would you consult with respect to reproduction | RADGR47 | Consult with vet. practitioner | 590 | 44 | 0.2244 |
|  |  | Consult others or none | 1210 | 45 |  |
| Who would you consult with respect to disease prevention | RADGP48 | Consult with vet. practitioner | 834 | 43 | 0.2349 |
|  |  | Consult others or none | 966 | 45 |  |
| Antibiotic dry cow treatment. | GOLDA_50 | No | 261 | 32 | <.0001 |
|  |  | Several | 1194 | 46 |  |
|  |  | All | 345 | 49 |  |
| **Owners appraisal** |  |  |  |  |  |
| Intensity of cow house | BELAEG65 | Too intensive | 414 | 47 | 0.0259 |
|  |  | Not intensive enough | 161 | 40 |  |
|  |  | Adequate | 1225 | 44 |  |
| How satisfied with cow health | SUNDH_51 | Less satisfied | 90 | 64 | <.0001 |
|  |  | Medium | 453 | 52 |  |
|  |  | Satisfied | 981 | 42 |  |
|  |  | Very satisfied | 276 | 32 |  |
| How satisfied with cow welfare. | VELF_52 | Less satisfied | 23 | 54 | 0.0137 |
|  |  | Medium | 244 | 47 |  |
|  |  | Satisfied | 1080 | 45 |  |
|  |  | Very satisfied | 453 | 42 |  |
| Diagnostic criteria for CM | SLEMT_57 | No answer | 262 | 41 | <.0001 |
|  |  | Changed milk | 720 | 48 |  |
|  |  | Changed milk & gland | 818 | 42 |  |

(1) MILK: Mastitis incidence rate was estimated to increase by 1.24 cases per 1 Kg milk production per day.

(2) MGRAES27: Mastitis incidence rate was estimated to decrease by 0.31 cases per 1 month of pastured.

(3) UKLOV_43: Mastitis incidence was estimated to increase by 0.35 cases per 1% increase of claw diseases.
